# Effects of Age, Drug Dose, and Sampling Time on Salivary Levels of Olanzapine, Quetiapine, and Their Metabolites

**DOI:** 10.3390/jcm9103288

**Published:** 2020-10-13

**Authors:** Ewelina Dziurkowska, Marek Wesołowski

**Affiliations:** Department of Analytical Chemistry, Medical University of Gdansk, Gen. J. Hallera 107, 80-416 Gdansk, Poland; marek.wesolowski@gumed.edu.pl

**Keywords:** saliva, olanzapine, quetiapine, metabolites, therapeutic drug monitoring, patient-related variables

## Abstract

Although blood is the basic test material to monitor levels of antipsychotic drugs in a person’s system, saliva could serve as a more convenient test material. Therefore, the aim of this novel study was to determine the correlations between the salivary levels of olanzapine and quetiapine (and their metabolites: N-demethyl olanzapine and norquetiapine) and the patient’s sex and age, dose level, and the time of sampling. The study involved two groups of patients: 21 female patients starting treatment immediately after being admitted to the hospital and 36 male and female nursing home residents, long-time users of the studied drugs. Women had lower levels of the tested analytes than men. Quetiapine levels in the saliva of people starting the treatment showed a positive correlation with the age of the patients and a strong positive correlation with the dose level. The saliva levels of olanzapine showed a strong correlation with its metabolite in patients who had recently started treatment. Among long-time users of this drug, salivary levels differed significantly before and after administration. In conclusion, the results indicate that there is a possibility of using saliva as a material for monitoring quetiapine or olanzapine concentrations, especially in people starting treatment.

## 1. Introduction

Olanzapine and quetiapine belong to the second-generation of antipsychotics, the most frequently applied drugs in personality disorders. Oral bioavailability of olanzapine is 60% and the highest blood concentration after oral administration is reached within 6 h. For quetiapine, the values are 9% and 1.5 h, respectively. Both drugs give a quick healing effect and improve the patient’s emotional state and cause a smaller number of side effects than the previous generations of antipsychotics. However, due to the frequent use of polypharmacy and the high risk of side effects when maximum doses are used [1], it is advisable to constantly monitor both side effects and drug concentrations in patients. Therapeutic drug monitoring (TDM) allows adjusting the dose of the drug to the individual needs of the patient, avoiding severe side effects or drug interactions. Indications that the treatment of antipsychotic drugs should be monitored include a lack of cooperation between the patient and the attending physician, lack or insufficiency of clinical response at normal doses, as well as in the treatment of children, adolescents, and elderly patients [2]. However, it should be remembered that levels of the drug in the blood may vary not only according to the dose used, but also the sex, age, or weight of the patient.

Research on the relationship between drug levels in the body and the dose, weight, age, or levels of additional drugs in the system is usually conducted in the blood. It has been shown that in women, as well as in people over 60 years of age, the concentration of olanzapine in the blood is higher than in men and younger people [3,4,5,6,7,8,9]. It was also noted that in smokers, the metabolism of olanzapine is faster and the drug concentration is lower than in non-smokers [3,4,6,7,10,11]. Moreover, olanzapine shows very high variability of its concentration in blood, reaching as much as 47% [3,12], which was found when serum samples were collected multiple times (up to 24) from the same person, treated with the same dose, over a period of several months.

Similarly, quetiapine shows higher drug concentrations in women than in men [8,9,13,14]. Age is another factor that prolongs its metabolism, resulting in higher quetiapine levels in individuals over 65 years of age [8,9,13,14]. 

The concentrations of olanzapine and quetiapine in blood also depend on the applied dose. Quetiapine has been shown to have a weak correlation between the dose and the blood concentration [15,16,17,18]. However, the quetiapine’s metabolite, N-desalkylquetiapine, is more strongly correlated with the dose than the parent compound [15]. In patients treated with olanzapine, the drug’s concentration in blood shows a linear correlation with the applied dose [3].

All studies on the relationships between the dose, age, or sex of the patient and olanzapine and quetiapine levels have been carried out in the blood. This approach allows us to determine the total concentration of the substance in the body of both the free and the protein-bound fraction. However, only a free fraction is responsible for the compound’s activity and is subject to metabolism. Therefore, the simultaneous use of other drugs metabolized by the same isoenzymes will be visible in the form of fluctuations in the concentration of the main metabolite of the analyzed drug. In addition, the rate of metabolism of the compound is indicated by the metabolite’s level. Both substances, free fraction and metabolite, are present in saliva. Usually, there is a correlation between the drug concentration in blood and saliva, and there is one available study that confirms it for quetiapine [19,20].

Blood collection is more cumbersome and requires the presence of qualified personnel. There are no problems with taking saliva. The patient may sample their saliva themselves at a specific time of day and then take it for analysis. In addition, saliva collection, which does not require needles, is much less stressful to patients who are then more frequently willing to participate in sampling. Determination of the concentration of drugs in saliva is also recommended for children. In this age group, the use of quetiapine and olanzapine is often associated with an improper dosage of drugs resulting in excessive olanzapine and insufficient quetiapine levels in the body [21]. This may cause an increase in side effects or a lack of response to the treatment.

Given the above, the aim of the study was to determine the relationship between the concentration of the drug determined in saliva and the dose, age, and sex of patients. The results obtained will be used to assess the possibility of using saliva as a biological material, allowing to monitor the concentration of drugs in the body.

## 2. Experimental Section

### 2.1. Study Participants

The study included 21 (F-female) patients of the Nervous and Mentally Ill Hospital in Starogard Gdanski and 36 (M-male and F-female) residents of the Nursing Home in Damaszka. Volunteers were included in the study on the basis of a medical interview. Among the enrolled were people suffering from schizophrenia and bipolar disorder. The saliva concentrations of analytes (olanzapine or quetiapine and their metabolites) were determined in two groups of subjects. The saliva concentrations of analytes (olanzapine or quetiapine and their metabolites) were determined in two groups of subjects. The first group consisted of women starting treatment with quetiapine or olanzapine (on the day of admission to the hospital), the second group (men and women) were those who were receiving long-term treatment (for at least several years). Both groups of patients were given drugs orally, and the average daily dose is presented in Table 1. The analytes were sampled for 14 days, 15 min before drug administration and 2 h after morning application in those having long-term treatment, in order to determine fluctuations in the concentration of the substance between its applications. 

In contrast, in those who were starting the treatment, the concentration of analytes was determined only 2 h after drug administration (given once a day at the same time), every day for a period of 14 days, to determine whether the concentration of analytes changed or stabilized over several days and reached a value that persisted for a long time. The study was also used to determine any differences in the concentration of analytes between persons entering treatment and those using the drugs for a long time. In addition to determining the levels of quetiapine, olanzapine, and their metabolites, information about the dose used, the age of the patients, and their sex were also collected, and are presented in detail in Table 1.

Saliva samples were collected from the patients without stimulation, by direct placement in a plastic test tube. Details on sample collection and storage have been published in our previous paper [22]. The research project was approved by Ethical Committee at the Medical University of Gdansk, Poland (NKBBN/139/2016).

### 2.2. Drug Assays

The concentration of compounds in saliva was determined using UHPLC (Ultra High Performance Liquid Chromatography) with DAD (diode array) detection, following their isolation using solid phase extraction [23]. First, shortly after collection from patients, the saliva samples were frozen and stored at −21 °C until analysis. Thawed saliva was centrifuged and then 1 mL samples were transferred to polypropylene tubes, to which 1 mL of 2% FA (formic acid) and 1 mL of methanol/water (1:1) was added. The tubes were then shaken and centrifuged. The supernatant was loaded onto activated Strata-X-C columns. After washing with 2 mL of water and a 2 mL mixture of water and methanol (1:1), sorbent was dried for 10 min and adsorbed analytes were eluted using a 5% solution of ammonia in methanol. The eluate was dried and the dry residue was dissolved in 100 µL of the mobile phase at a 90:10 (v/v) proportion of solvent A (water with FA, pH 3.5) with the addition of 0.1% trimethylamine (TEA) to solvent B (acetonitrile). Then, 10 µL of the sample was injected onto a Luna Omega 3 µm column (LC Column 150 × 3.0 mm; Phenomenex, Torrance, CA, USA) with Polar C18 100 filling and a precolumn (Polar C18, 4 × 2.0 mm ID; Phenomenex, Torrance, CA, USA).

A UHPLC Nexera XR liquid chromatograph equipped with a CBM-20Alite control system, SIL-30AC autosampler, LC-30AD pump, CTO-20AC thermostat and UV-VIS SPD-M30A detector with matrix diode and high-sensitivity measuring cell SPD-M30A (85 mm) (Shimadzu, Kyoto, Japan) was used for chromatographic separation. The analytes were eluted by the mobile phase with a flow rate of 0.6 mL/min with a gradient program starting from an 85:15 (v/v) proportion of solvent A to solvent B, to a 35:65 (v/v) proportion of solvent A to solvent B. The temperature of the thermostat showed 35 °C.

The method allows for the simultaneous determination of five antipsychotic drugs with metabolites as well as carbamazepine and carbamazepine—10,11 epoxide. The intra-day precision for olanzapine ranged from 2.33–9.90%. In the case of N-demethyl olanzapine, between 2.01 and 6.76%. On the other hand, for quetiapine and the metabolite, CV values were 3.91–5.88% and 4.98–7.25%, respectively. CV values for inter-day precision were determined to be 7.49–17.27%, 7.68–13.93%, 5.35–13.93% and 8.22–14.60% for olanzapine, N-demethyl olanzapine, quetiapine and norquetiapine, respectively.

### 2.3. Statistical Analysis

The concentration of olanzapine and quetiapine and their metabolites was determined both in raw and dose-corrected values. The raw concentration value of each of the analytes was determined as the average concentration measured in a given person over the time of the study. The mean value was then divided by the daily dose of the drug to obtain dose-corrected values. It was also examined whether there are differences in analyte levels depending on the time elapsed from drug administration to sampling, whether these differences apply only to the parent substances or also to metabolites and whether they are statistically significant. Then, the mean value was calculated from the raw value in order to make the concentration independent of the time of sampling, both for the parent compound and its metabolite. The analysis concerned the correlations between drug levels and gender and age, with the significance of differences determined using the Spearman rank coefficient. Statistically significant differences in the concentration of olanzapine and its metabolite between the different groups were determined using a non-parametric two-tailed Mann-Whitney U-test or Wilcoxon test. The significance level was set at *p* < 0.05 (Statistica 13.3).

## 3. Results

In total, 798 saliva samples from 57 people (24 F and 33 M) were tested to determine the concentration of olanzapine and its metabolite (12 F and 20 M) and quetiapine and its metabolite (12 F and 13 M). Samples were taken once a day from people starting treatment—about 2 h after drug administration for 14 days. In the case of people regularly treated with olanzapine or quetiapine for several years, samples were taken twice a day for 14 days—15 min before administration and about 2 h after its application. The results of the research and statistical analysis are presented in Table 2.

### 3.1. The Concentration of Olanzapine and N-demethyl olanzapine in Saliva

**Figure 1:**
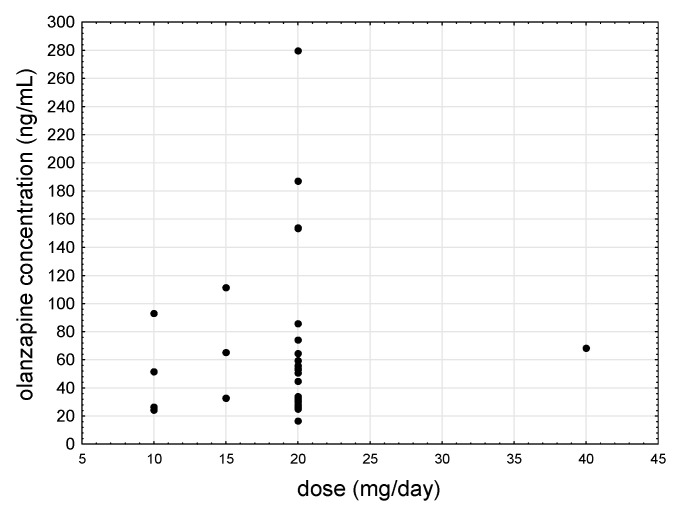
Scatter plot of all patients’ saliva mean olanzapine concentrations as a function of daily dose.

In subjects starting treatment (mean age ± SD was 52 ± 11 years), the mean concentration of olanzapine and its metabolite, N-demethyl olanzapine, (mean ± SD) was 32.3 ± 11.7 and 6.7 ± 3.7 ng/mL, respectively. In patients regularly treated with olanzapine (mean age ± SD was 54 ± 11 years), the mean concentration of the analytes in salivary samples taken 15 min before drug application was 57.9 ± 40.3 ng/mL (olanzapine) and 20.3 ± 11.8 ng/mL (N-demethyl olanzapine), and 2 h after the administration the respective levels were 112.9 ± 98.3 and 23.1 ± 16.1 ng/mL. Figure 1 shows the relationship between the daily dose of olanzapine and saliva concentration.

### 3.2. Correlation between Olanzapine and N-demethyl olanzapine Concentrations and Age, Gender and Dose

Statistical analysis showed that the mean olanzapine concentration in saliva samples before drug administration were statistically significantly lower than after the drug (*p* < 0.05, Wilcoxon test). The differences in the levels of its metabolite, N-demethyl olanzapine, were not statistically significant. 

Statistically significant differences were observed in the concentration of olanzapine between samples taken from patients starting treatment with olanzapine (in the latter group we took into account the concentrations determined after the administration of the drug) (Mann-Whitney U-test). Then, the concentration of olanzapine in the saliva of patients starting treatment and continuing in the samples taken before drug administration was compared, considering only the marked concentration of the compound. Additionally, in the case of the concentration, differences were statistically significant. A similar relationship was found when the active metabolite, N-demethyl olanzapine (*p* < 0.05), was determined. Dose-corrected values also showed statistically significant differences in the concentrations of both analytes between patients entering and continuing treatment.

**Figure 2:**
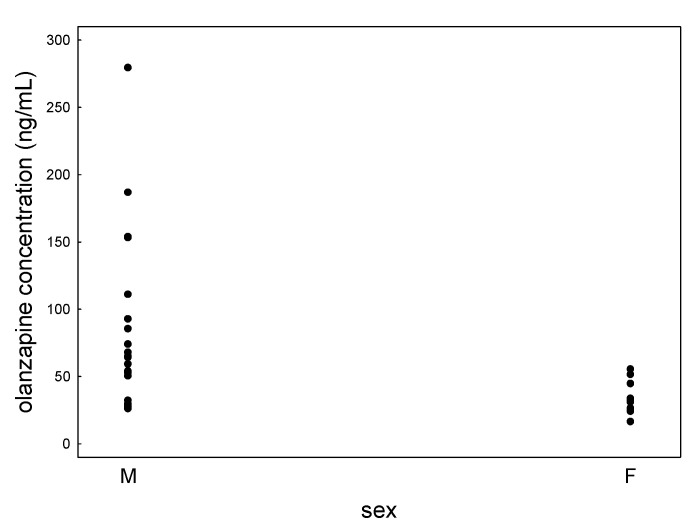
Scatter plot of olanzapine concentrations determined in females’ (F) and males’ (M) saliva.

The mean olanzapine concentration in women was statistically significantly lower than in men — 33.4 ± 11.7 compared to 86.1 ± 63.6 ng/mL (Figure 2). The use of dose-corrected values confirmed the statistically significant difference between sexes.

Statistical analysis of olanzapine and N-demethyl olanzapine in patients over 60 years of age and 70 years of age showed no significant differences in both raw values and dose-corrected values.

On the basis of Spearman’s rank order correlation, a strong negative correlation between sex and mean concentration of olanzapine and N-demethyl olanzapine was found (r = −0.6082 and r = −0.6012). Similarly, when using dose-corrected values, the correlation was also significant (r = −0.5383 for olanzapine and r = −0.5593 for the metabolite).

The age of the patients was strongly negatively correlated with the average concentration of the olanzapine metabolite in saliva collected from patients two hours after drug administration (the group of long-time users).

**Figure 3:**
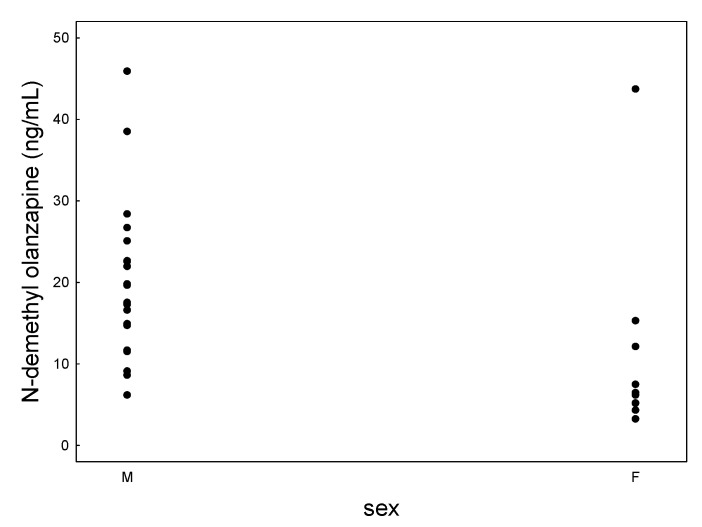
Scatter plot of N-demethyl olanzapine concentrations determined in females’ and males’ saliva.

The mean concentration of N-demethyl olanzapine determined in women was 9.8 ± 11.3 ng/mL, compared to 20.0 ± 9.8 ng/mL in men (Figure 3). The Mann-Whitney U test showed significant statistical differences for dose-corrected values.

**Figure 4:**
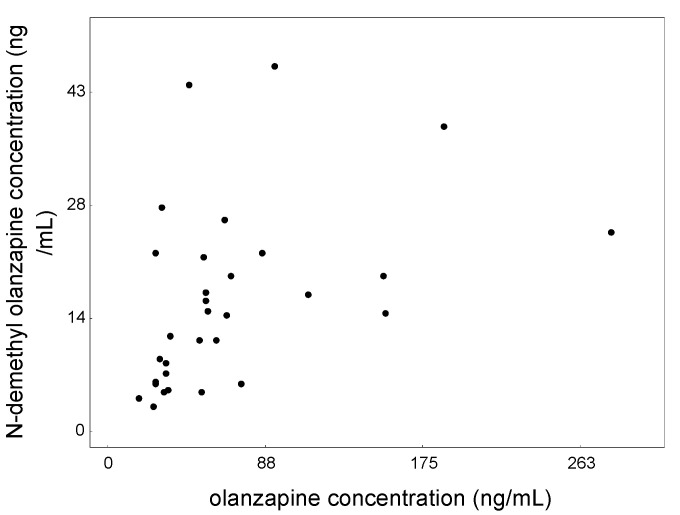
Relationship between mean olanzapine concentration and mean N-demethyl olanzapine concentration determine in all saliva patients treated with olanzapine.

A strong positive correlation was observed between the mean concentration of olanzapine and its metabolite for the entire population (Figure 4), r = 0.5880 and in the group starting treatment (r = 0.7091). A high positive correlation was also found between the mean olanzapine concentration (raw values) and (i) dose-corrected values of olanzapine (r = 0.8911) and (ii) dose-corrected values of its metabolite (r = 0.5469).

The mean concentration of N-demethyl olanzapine showed an additional strong positive correlation with dose-corrected values of olanzapine and N-demethyl olanzapine, r = 0.5169 and r = 0.9322, respectively.

In people entering treatment, there was a strong positive correlation between the mean concentration of olanzapine (raw values) and the dose-corrected values of olanzapine (r = 0.7273) and between the concentration of olanzapine (raw values) and N-demethyl olanzapine (r = 0.8364, respectively).

### 3.3. Quetiapine and Norquetiapine Concentration in Saliva

The mean quetiapine and norquetiapine levels in patients who were starting the treatment (mean age ± SD was 49 ± 7 years) were 51.8 ± 61.3 ng/mL and 25.2 ± 15.8 ng/mL, respectively. In patients who were constantly treated with quetiapine (mean age ± SD was 49 ± 11 years), the mean concentration of analytes in saliva samples taken 15 min before drug application was 110.4 ± 126.9 ng/mL for quetiapine and 54.3 ± 48.3 ng/mL for norquetiapine. Analysis of saliva samples obtained 2 h after drug administration showed the mean quetiapine level at 115.1 ± 137.8 and norquetiapine at 57.6 ± 76.9 ng/mL.

### 3.4. Correlation between Quetiapine and Norquetiapine Concentration and Age, Gender and Dose

Statistical analysis showed no statistically significant differences in the mean concentration of quetiapine and its metabolite between saliva samples obtained before and after drug administration (*p* < 0.05, Wilcoxon test). Comparing the average concentration of quetiapine in saliva samples of patients entering treatment and those continuing it, the latter group showed statistically significantly higher levels after the administration of the drug. There was no statistically significant difference for its metabolite, norquetiapine (*p* < 0.05). Dose-corrected values also did not show statistically significant differences in the concentrations of both analytes between patients entering and continuing treatment.

**Figure 5:**
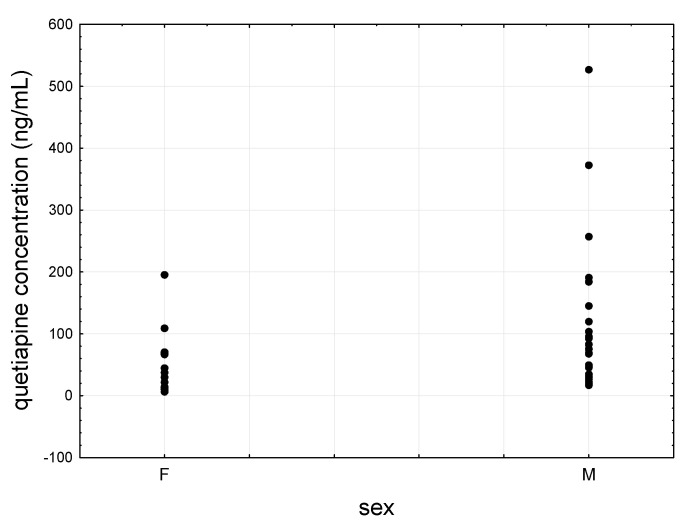
Scatter plot of quetiapine concentrations determined in females’ (F) and males’ (M) saliva.

**Figure 6:**
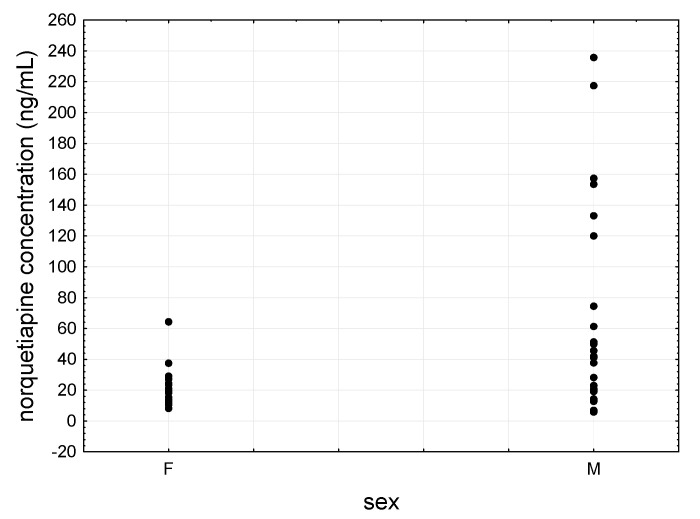
Scatter plot of norquetiapine concentrations determined females’ (F) and males’ (M) saliva.

Quetiapine showed lower levels in women than in men, i.e., 50.6 ± 55.7 ng/mL vs. 123.2 ± 105.1 ng/mL (Figure 5), although the difference was statistically significant only for raw values. For norquetiapine, whose mean concentration in women was 24.00 ng/mL ± 14.6 ng/mL and in men 61.8 ± 58.9 ng/mL (Figure 6), the difference was not statistically significant for both raw and dose-corrected values. 

The concentrations of quetiapine and norquetiapine were statistically significantly higher for people over 40 years of age in relation to those below 40.

**Figure 7:**
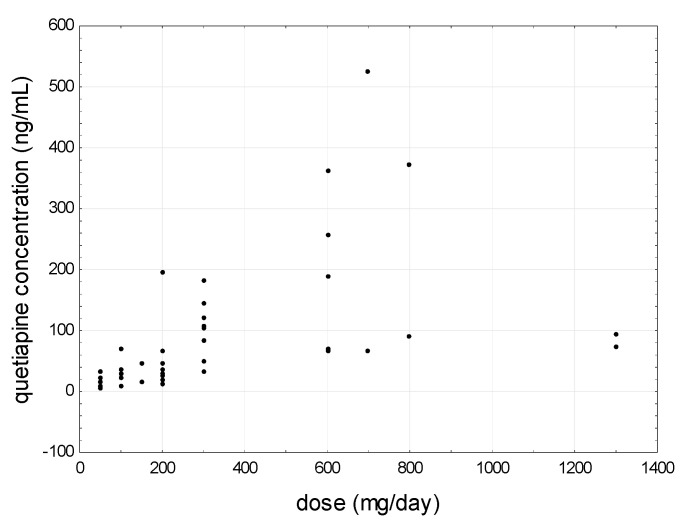
Scatter plot of all patients’ saliva mean quetiapine concentrations as a function of daily dose.

**Figure 8:**
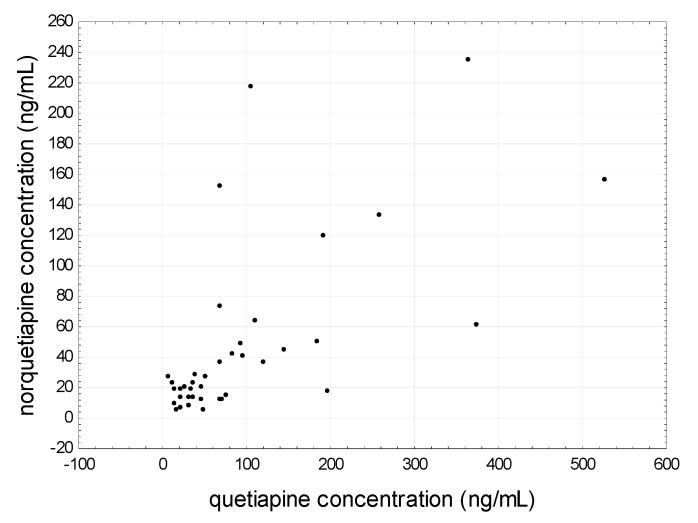
Relationship between mean quetiapine concentration and mean norquetiapine concentration determine in saliva patients treated with quetiapine.

Spearman’s rank correlation coefficient showed that mean quetiapine levels strongly positively correlated with the dose (r = 0.7568) (Figure 7) and the mean concentration of norquetiapine (r = 0.6492) (Figure 8). In addition, in the saliva of patients entering treatment, the average concentration of quetiapine strongly positively correlated with the average concentration of norquetiapine (r = 0.9030) and the dose of the drug used (r = 0.6138). A negative correlation was found between norquetiapine and the age of patients (r = −0.4070). Analysis of quetiapine concentration in saliva taken 15 min before drug administration showed a strong positive correlation with norquetiapine determined in the same saliva samples (r = 0.8357). In addition, norquetiapine concentration determined in samples before drug administration strongly positively correlated with the metabolite determined two hours after the administration of quetiapine (r = 0.8893). Among women, a strong positive correlation was noted between the mean concentration of quetiapine (raw values) and dose-corrected values (r = 0.6396). Among men, a strong positive correlation was observed between the raw averaged values of quetiapine (before and after administration) and the concentration of this compound in the saliva taken before drug application (r = 0.6264).

## 4. Discussion

As a diagnostic material, saliva can be used as an indicator of mental health. Endogenous compound levels such as cortisol and alpha-amylase may play an important role as biomarkers of stress, depression and anxiety disorders [24]. In contrast, in patients with schizophrenia, bipolar and ADHD, genetic changes were observed in four genes [25]. On the other hand, determination of drugs and metabolites in saliva allows for monitoring their concentration and, consequently, individualization of therapy.

Previous studies on the dependence of olanzapine or quetiapine on the dose, sex or age of patients have been conducted only through the blood. However, given the inconvenience associated with its extraction, it is advisable to look for alternative biological materials, and thus, the aim of this study was to determine the relationships between sex, patient age, drug dose and time of sampling, and the concentration of olanzapine, quetiapine and their major metabolites in saliva. The study involved the analysis of olanzapine, quetiapine and their metabolites (N-demethyl olanzapine and norquetiapine, respectively) in 798 saliva samples from both sexes.

Our results showed that olanzapine and quetiapine had lower concentrations in the saliva of women. This sex-related difference was also observed for olanzapine when using dose-corrected values—these were also lower in women. Raw levels of its metabolite, N-demethyl olanzapine, were also lower in women. However, literature data show that their blood levels are higher in women than in men [3,4,5,6,7,8,9,14,15], which may be associated with a certain limitation of our study, where the group starting the treatment consisted solely of women, and the group of long-time users was predominantly male. 

After adjusting for the dose, quetiapine levels in women did remain lower, but the differences were not statistically significant. Literature data do not indicate whether the differences in blood quetiapine levels in men and women are statistically significant, but usually the concentration in women is higher, although not in all cases [14]. 

Available literature data rarely describe the analysis of olanzapine and quetiapine together with their metabolites, the level of which could indicate their rate of metabolism and possible interactions with other substances. There is only one available publication that shows that blood norquetiapine levels are not statistically significantly dependent on sex [15].

The study also aimed to determine the relationship between patient age and analyte concentration. For olanzapine, the age of the patients affected neither the level of the parent compound nor its metabolite. Except for N-demethyl olanzapine measured in saliva taken 2 h after drug administration from patients who continued treatment. Some literature data indicate a decrease in olanzapine metabolism in patients over the age of 60 years [3,4,8]. However, other authors indicate a relatively weak relationship between age and olanzapine levels [8]. 

In our study, all samples came from smokers, and literature data indicate a faster metabolism of olanzapine and a lower concentration of the compound in the smokers’ body [3,4,10,11]. 

There is no available information in the literature on the effects of smoking on the metabolism of quetiapine. This research indicates that the concentrations of both the parent compound and metabolite were higher in the saliva of people over 40. This observation is in line with literature reports on blood levels [8,13].

Literature reports emphasize the high individual variability of olanzapine blood concentrations [3,9,12]. This study showed a small correlation between the dose and concentration of this analyte in saliva, which can be explained by this high individual variation. At the same time, however, a high correlation was observed between the concentration of the parent compound and its metabolite, the strongest correlation being for those entering treatment (Spearman r = 0.7091). This means that the simultaneous determination of olanzapine and N-demethyl olanzapine can be used to monitor the metabolism of the parent compound in this group of patients. Analysis of olanzapine and metabolite levels in a given patient in the following days (the group entering the treatment) also showed a significant correlation between analytes, with r = 0.7 at *p* < 0.05.

A comparison of saliva olanzapine levels collected 15 min before and 2 h after application of the drug show a considerable and statistically significant difference which was not observed for its metabolite, N-demethyl olanzapine. This observation is in line with literature data, which indicate the significance of time elapsed from the application of the drug to the collection of samples for the determined blood drug levels [7].

Blood quetiapine levels are known to weakly correlate with the dose of the drug [16,17,18], although Spearman’s rank correlation in our study showed a strong positive correlation between the dose and the concentrations of quetiapine and its metabolite in saliva. This strong correlation may indicate a potential benefit of determining and monitoring changes in saliva quetiapine levels. Some literature data indicate a correlation of quetiapine concentration in saliva and blood, which indicates that saliva could be used to monitor quetiapine levels and its metabolisms [19,21].

This study showed no strong fluctuations in analyte levels in patients continuing quetiapine treatment. Comparing the concentrations determined before and after drug administration, no statistically significant differences were noticed. However, such differences were observed between the patients entering and continuing the treatment, with the latter group showing much higher levels, which suggests that optimal and stable levels of the drug in the patient’s body can be achieved only in long-term administration. Such a relationship was not observed with norquetiapine, which may be due to faster metabolism of the parent substance compared to the metabolite, which confirms the literature report on the lower pharmacokinetic variability for blood norquetiapine levels compared to its parent compound [14].

The study has several limitations. First of all, the patients starting the treatment are women, while the patients continuing it are mostly men. The study showed that the concentration of both drugs in saliva of women was lower. On the one hand, this shows differences in terms of sex, but also shows that the concentration in the case of people starting the treatment is lower. Therefore, in order to clarify whether the differences in the concentrations of the parent compounds are due to the effect of sex on drug metabolism or due to the duration of the treatment, the analyzed substances should be determined in the saliva of men starting treatment in the next stage of the study.

The secretory efficiency of the salivary glands as well as the antipsychotic drugs used may affect the amount of saliva secreted and its composition. At enrollment in the study, no testing for function of the salivary glands was performed. In any case, the examined patients did not show problems with dry mouth. However, if these problems arise, using cotton swabs for the sampling should be considered, as that would facilitate the process.

Another important aspect is the observation of a correlation between the concentration of quetiapine and its metabolite in saliva. The literature data indicate only a weak correlation between the measured concentration and the dose of the drug. However, the time between the administration of quetiapine and the sampling was 7 h [16] or 10–12 h [17], but the time when quetiapine reaches its highest concentration in the blood after oral administration is 1.5 h [1] or even 2 h [16]. Accordingly, a significant part of the drug was already metabolized. Sampling 2 h after drug application allowed to find the correlation between dose and concentration. Therefore, in order to confirm the obtained results, similar studies with more volunteers will be performed in the next time.

The literature data show that the highest concentration of olanzapine in the blood is reached after approximately 6 h. In this study, saliva samples were collected 15 min before and 2 h after drug administration. This slight time difference showed that even here the differences between the pre- and post-concentration were statistically significant. Determining the level of olanzapine before application and when it reaches the highest concentration in the blood may confirm statistically significant differences.

The majority of the patients were taking other medications beside olanzapine or quetiapine. However, the individual additional medications were administered to a small number of subjects, and therefore, it is not possible to draw conclusions about their effects. In addition, nursing home residents are an isolated population. This made it possible to exclude the influence of consumed food, drinks and lifestyle on the results obtained. On the other hand, all these factors should also be taken into account, which would reflect the transfer of insights to a more diverse population.

## 5. Conclusions

The study was aimed at determining the impact of the patient’s age, sex, time of sampling and the dose of drug used, on its concentration in the body determined in the saliva of 47 people of both sexes treated with olanzapine or quetiapine. The concentration of parent compounds together with metabolites was analyzed in the samples. A lower concentration of all analyzed compounds was found in the saliva of women than men. In addition, a significant effect of patient age on the level of quetiapine was observed, as well as weaker fluctuations in quetiapine in patients starting the treatment compared to its metabolite. There was also a strong correlation between the dose used and the parent compound concentration in the group starting the treatment. In addition, the strong relationship between quetiapine and norquetiapine concentrations determined in patient samples 15 min before drug application (in long-term treatment) allows the monitoring of quetiapine metabolism. In the case of olanzapine, such a possibility exists in persons starting treatment due to the strong correlation between the parent compound and the metabolite determined in the same sample. Olanzapine levels also showed a strong effect of time between sampling and application of the drug. This shows the importance of standardized time between application and saliva sampling, as it may strongly affect the determinations.

The results obtained are partly consistent with literature reports on blood levels, which indicate the possibility of using saliva as a biological material for monitoring the concentration of olanzapine and quetiapine together with metabolites in patients. However, further research on a larger group of patients is required.

## Figures and Tables

**Table 1:** Patients characteristics. The demographic date and daily dose of both sexes, male (M) and female (F), treated with olanzapine (32) or quetiapine (25).

Drug		Olanzapine	Quetiapine
Patients		*n* 32 (12 F; 20 M)	*n* 25 (12 F; 13 M)
Age (year)	mean	53.7	49.9
	median	55.5	50
	range	21–74	31–64
Dose (mg/day)	mean	18.9	314
	median	20	200
	range	10–40	50–1300

**Table 2:** Research results regarding the effect of age, sex, dose used and time of sampling on the concentration of olanzapine, quetiapine and metabolites.

Dependent Variables		Range	Mean ± SD	%Difference *	Mann-Whitney U-Test; *p*
Mean olanzapine saliva concentration (ng/mL)	Female	16.5–55.7	33.4 ± 11.7	157.8	<0.001
	Male	26.4–279.5	86.1 ± 63.6		
Mean N-demethyl olanzapine saliva concentration (ng/mL)	Female	3.3–43.7	9.8 ± 11.3	104.1	<0.001
	Male	6.2–45.9	20.0 ± 9.8		
Mean olanzapine saliva concentration/dose (ng/mL)/(mg/day)	Female	0.8–2.8	1.9 ± 0.7	152.6	0.003
	Male	1.3–14.0	4.8 ± 4.0		
Mean N-demethyl olanzapine saliva concentration/dose (ng/mL)/(mg/day)	Female	0.2–2.2	0.5 ± 0.6	120.0	0.002
	Male	0.3–2.3	1.1 ± 0.7		
Olanzapine saliva concentration (ng/mL)	1. 15 min before application	21.0–169.8	57.9 ± 40.3	Between 1&2 48.7 ** 1&3 44.2 *** 2&3 71.4 **	0.043 0.032 <0.001
	2. 2 h after application	23.6–396.7	112.9 ± 98.3		
	3. Starting treatment	16.5–55.7	32.3 ± 11.7		
N-demethyl olanzapine saliva concentration (ng/mL)	1. 15 min before application	7.2–53.1	20.3 ± 11.8	Between 1&2 12.1 ** 1&3 71.0 *** 2&3 70.0 **	0.873 <0.001 <0.001
	2. 2 h after application	4.6–63.9	23.1 ± 16.1		
	3. Starting treatment	3.3–15.3	6.7 ± 3.7		
Mean quetiapine saliva concentration (ng/mL)	Female	6.4–195.3	50.6 ± 55.7	143.5	0.027
	Male	23.1–326.6	123.2 ± 105.1		
Mean norquetiapine saliva concentration (ng/mL)	Female	10.5–64.3	24.0 ± 14.6	157.5	0.067
	Male	9.2–177.8	61.8 ± 58.9		
Mean quetiapine saliva concentration/dose (ng/mL)/(mg/day)	Female	0.1–1.0	0.3 ± 0.3	133.3	0.155
	Male	0.1–2.9	0.7±0.7		
Mean norquetiapine saliva concentration/dose (ng/mL)/(mg/day)	Female	0.05–0.5	0.2 ± 0.2	0.0	0.755
	Male	0.02–0.4	0.2 ± 0.1		
Quetiapine saliva concentration (ng/mL)	1. 15 min before application	26.1–526.4	110.4 ± 126.9	Between 1&2 4.1 ** 1&3 53.1 *** 2&3 55.00 **	0.326 0.135 0.0306
	2. 2 h after application	17.1–462.4	115.1 ± 137.8		
	3. Starting treatment	6.4–195.3	51.8 ± 61.3		
Norquetiapine saliva concentration (ng/mL)	1. 15 min before application	12.6–157.4	54.3 ± 48.3	Between 1&2 5.7 ** 1&3 53.6 *** 2&3 56.3 **	0.320 0.524 0.524
	2. 2 h after application	5.8–235.6	57.6 ± 76.9		
	3. Starting treatment	10.5–64.2	25.2 ± 15.8		

* The mean value of female was used as reference (100%). ** The mean value of concentration determined in saliva samples 2 h after application of the drug (2). *** The mean value of concentration determined in saliva samples 15 min before application of the drug (1).
